# The role of the *C. albicans* transcriptional repressor *NRG1* during filamentation and disseminated candidiasis is strain dependent

**DOI:** 10.1128/msphere.00785-23

**Published:** 2024-02-20

**Authors:** Rohan S. Wakade, Melanie Wellington, Damian J. Krysan

**Affiliations:** 1 Department of Pediatrics, Carver College of Medicine, University of Iowa, Iowa City, Iowa, USA; 2 Department of Molecular Physiology and Biophysics, Carver College of Medicine, University of Iowa, Iowa City, Iowa, USA; Duke University Hospital, Durham, North Carolina, USA

**Keywords:** *Candida albicans*, hyphae, fungal pathogenesis

## Abstract

*Candida albicans* is one of the most common causes of superficial and invasive fungal diseases in humans. Its ability to cause disease is closely linked to its ability to undergo a morphological transition from budding yeast to filamentous forms (hyphae and pseudohyphae). The extent to which *C. albicans* strains isolated from patients undergo filamentation varies significantly. In addition, the filamentation phenotypes of mutants involving transcription factors that positively regulate hyphal morphogenesis can also vary from strain to strain. Here, we characterized the virulence, *in vitro* and *in vivo* filamentation, and *in vitro* and *in vivo* hypha-associated gene expression profiles for four poorly filamenting *C. albicans* isolates and their corresponding deletion mutants of the repressor of filamentation *NRG1*. The two most virulent strains, 57055 and 78048, show robust *in vivo* filamentation but are predominately yeast phase under *in vitro* hypha induction; the two low-virulence strains (94015 and 78042) do not undergo filamentation well under either condition. *In vitro*, deletion of *NRG1* increases hyphae formation in the SC5314 derivative SN250, but only pseudohyphae are formed in the clinical isolates. Deletion of *NRG1* modestly increased the virulence of 78042, which was accompanied by increased expression of hypha-associated genes without an increase in filamentation. Strikingly, deletion of *NRG1* in 78048 reduced filamentation *in vivo*, expression of candidalysin (*ECE1*), and virulence without dramatically altering establishment of infection. Thus, the function of the conserved repressor *NRG1* in *C. albicans* shows strain-based heterogeneity during infection.

**IMPORTANCE:**

Clinical isolates of the human fungal pathogen *Candida albicans* show significant variation in their ability to undergo *in vitro* filamentation and in the function of well-characterized transcriptional regulators of filamentation. Here, we show that Nrg1, a key repressor of filamentation and filament specific gene expression in standard reference strains, has strain-dependent functions, particularly during infection. Most strikingly, loss of *NRG1* function can reduce filamentation, hypha-specific gene expression such as the toxin candidalysin, and virulence in some strains. Our data emphasize that the functions of seemingly fundamental and well-conserved transcriptional regulators such as Nrg1 are contextual with respect to both environment and genetic backgrounds.

## INTRODUCTION

*Candida albicans* is a component of the human mycobiome and one of the most common causes of human fungal infections in both immunocompetent and immunocompromised people (1). The ability of *C. albicans* to undergo morphological transitions between round, budding yeast forms and the filamentous morphologies of pseudohyphae and hyphae has been strongly correlated with the ability of *C. albicans* to cause disease (2). Accordingly, this virulence trait has been studied extensively, leading to the identification of a large number of genes that affect *C. albicans* filamentous morphogenesis (3). The majority of these genes were identified by experimentation with a single strain background, SC5314, either directly or through work with its auxotrophic derivatives.

In recent years, interest in the characterization of clinical isolates of *C. albicans* has increased (4–6). Through this work, varying degrees of phenotypic heterogeneity have been identified particularly with respect to *in vitro* filamentation and biofilm formation. Studies of a set of 20 clinical isolates predominantly from bloodstream infections (7) have found that *in vitro* filamentation does not correlate well with virulence phenotypes in mouse models of invasive disease (4). A large study of commensal isolates of the GI tract also found a large amount of *in vitro* phenotypic diversity, whereas virulence phenotypes in an invertebrate model were quite consistent, regardless of *in vitro* phenotype (8). Furthermore, *in vitro* studies of the function of transcription factors have also revealed that the strain background can have profound effects on the function of factors such as *BCR1* and *NRG1* (6, 9). Here, we describe our investigation of the effect of strain background on the role of the transcriptional repressor Nrg1 during *in vitro* filamentation, *in vivo* filamentation, and disseminated candidiasis in a mouse model.

*NRG1* is a repressor of both filamentation and hypha-associated gene expression in *C. albicans*, and its deletion in SC5314 derivatives leads to constitutive pseudohyphae formation under non-inducing conditions *in vitro* (10, 11). At the initiation of normal *in vitro* filamentation, *NRG1* expression is reduced, and its gene product, Nrg1, is degraded (12). This inhibition of Nrg1 function is correlated with the expression of hypha-specific genes, many of which appear to be direct Nrg1 binding targets (11). In contrast, constitutive expression of *NRG1* blocks *in vitro* and *in vivo* filamentation and prevents the development of disease, but not organ infection, in a mouse model of disseminated infection (13). In contrast, reduced expression of *NRG1* leads to increases in *in vitro* and *in vivo* filamentation and disease in the mouse model.

Because of its profound effects on filamentation and virulence in the SC5314 background, we were interested to explore the effect of strain background on *NRG1* function. To do so, we deleted *NRG1* in four poorly filamenting clinical isolates and examined the effect of those mutations on *in vitro* and *in vivo* filamentations and gene expression, as well as on virulence in the mouse disseminated candidiasis model. Although *nrg1*∆∆ mutants have relatively similar phenotypes and effects on gene expression *in vitro* across these strains, there are profound strain-based differences during mammalian infection.

## RESULTS

### *NRG1* deletion mutants in poorly filamenting clinical isolates establish infection, but their effect on virulence is isolate dependent

**Fig 1:**
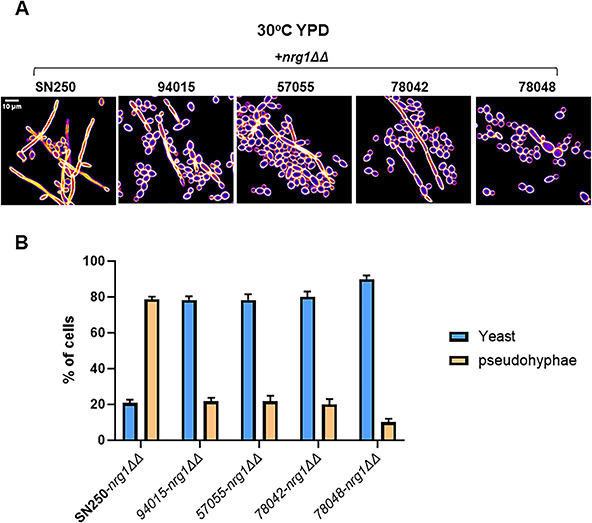
Morphology of *NRG1* deletion mutants in non-hypha-inducing conditions. (**A**) Representative images of *nrg1*∆∆ mutants in the indicated strain background after growth in yeast peptone dextrose medium in 30°C and calcofluor white staining. (**B**) Quantitation the yeast and pseudohyphal forms in the *nrg1*∆∆ mutants in the indicated strain backgrounds. The bars indicate the mean of three independent experiments with at least 100 cells counted in multiple microscopy fields. Error bars are standard deviations.

We selected four *C. albicans* clinical isolates [94015 (a/a), 57055 (a/α), 78048 (α/α), and 78042 (a/α)] that showed very low levels of filamentation after induction with Roswell Park Memorial Institute (RPMI) medium at 37°C as reported by Hirakawa et al. (4). Relative to SC5314 and its derivatives, all four strains were also shown previously to have reduced virulence in the mouse model of disseminated candidiasis (7). Hirakawa et al. found that 94015 contains a loss of function mutation in *EFG1*, which accounts for, at least part of, its reduced filamentation and virulence (7). None of the other three strains were found to have obvious loss of function mutations in other well-characterized regulators of filamentation (7). As expected, the *NRG1* deletion mutant in SN250 forms predominantly pseudohyphae in non-filament-inducing conditions (Fig. 1A and B). In contrast, the *nrg1*∆∆ mutants derived from the four clinical isolates are predominately in yeast phase in rich medium cultures at 30°C (Fig. 1A and B) and form approximately 20% pseudohyphae. On solid YPD medium at 30°C, the colony formed by the *nrg1*∆∆-SN250 mutant is wrinkled extensively, while *nrg1*∆∆ mutants in the other strains are much less wrinkled (Fig. S2A). Colony wrinkling generally correlates with pseudohypha formation.

**Fig 2:**
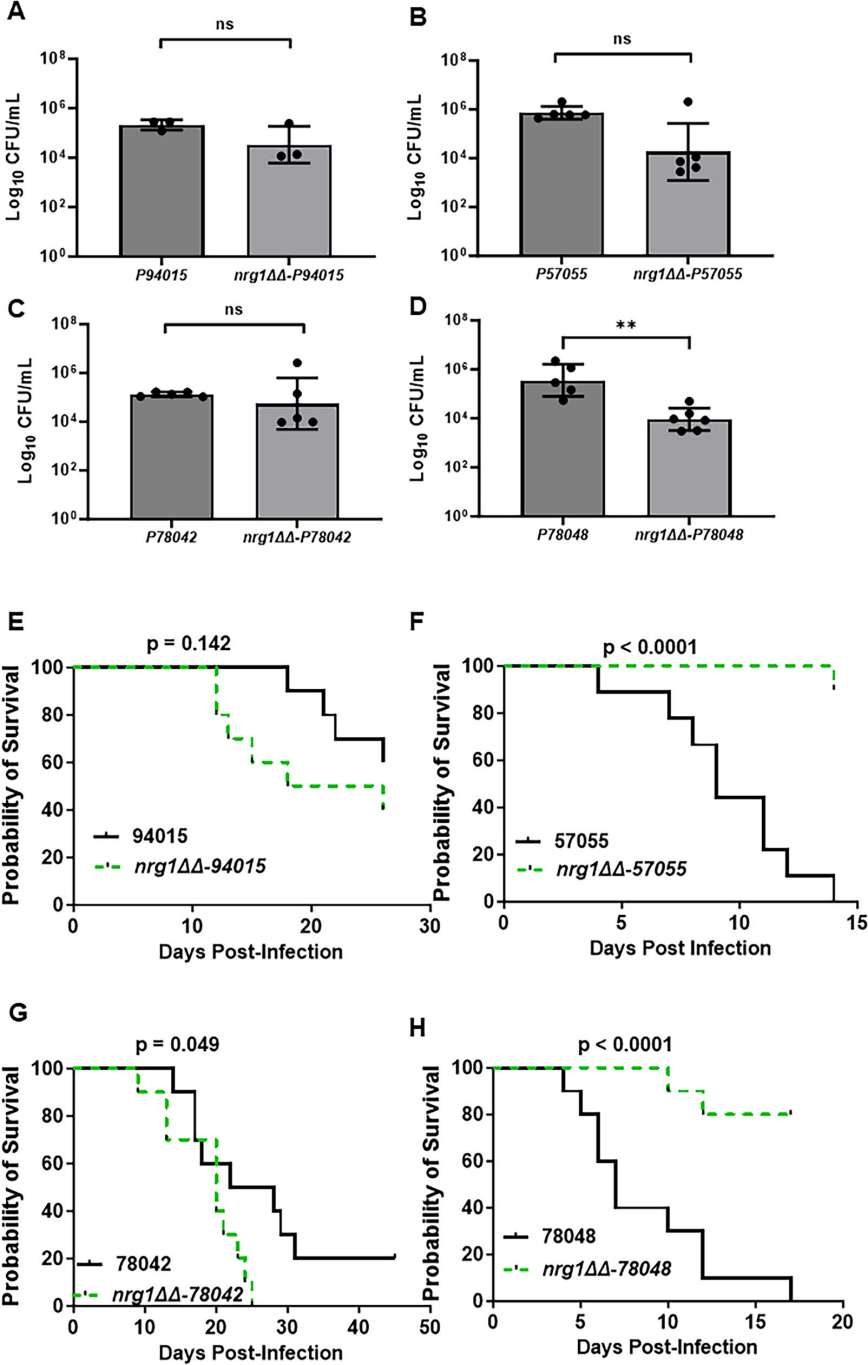
(**A**) *nrg1*∆∆ mutation has strain-dependent effects on infectivity and virulence. Kidney fungal burden 3 days post-infection for the indicated clinical strains (**A–D**) by tail vein and its corresponding *nrg1*∆∆ mutant (*n* = 5 mice per group) by tail-vein injection. Bars indicate mean of log_10_ fungal burden (CFU/mL) with individual mice shown as points and error bars indicating standard deviations. Differences between groups were analyzed by Student’s t test. ns indicates *P* > 0.05. *P < 0.05. Survival curves are shown for mice infected with the indicated clinical strains (**E–**H) and their corresponding *nrg1*∆∆ mutant (*n* = 10 mice per group) by tail-vein injection. The curves indicate survival defined as time to moribundity. *P* values are for log-rank (Mantel-Cox) analysis with statistical significance defined as *P* < 0.05. ns, not significant.

We inoculated outbred CD-1 mice with each of the four clinical isolates and their corresponding *nrg1∆∆* mutants and determined the kidney fungal burden at post-infection day 3. No mortality was observed prior to harvest, and all four parental strains established infections with between 10^5^ and 10^6^ colony-forming units (CFU)/mL of kidney homogenate (Fig. 2A through D). The corresponding *nrg1*∆∆ mutants also established infections. Mice infected with *nrg1*∆∆ mutants in the 94015 (Fig. 2A), 57055 (Fig. 2B), and 78042 (Fig. 2C) backgrounds showed no statistically significant difference in kidney burden relative to the parental strains (Student’s *t*-test of the log_10_-transformed data, *P* > 0.05), whereas mice infected with the *nrg1*∆∆−78048 strain showed reduced fungal burden relative to the parental stain (Fig. 2D). We did not test the SN250-derived *nrg1*∆∆ mutant because the extent of pseudohyphae formation precludes accurate inoculum quantitation.

Having confirmed that the parental strains and their corresponding *nrg1*∆∆ mutants established infection in the mouse model of disseminated candidiasis, we next compared their effect on virulence (Fig. 2E through G). Importantly, all clinical isolates caused some level of disease, with 57055 and 78042 causing moribundity [median survival times: 9 days (57055) and 7 days (78048)] comparable to previously reported data for SN250 in the same mouse strain background [median survival time: 6 days (SN250) (14)]. The strain lacking a functional allele of *EFG1* (4), 94015, was the least virulent, with only 40% moribundity 4 weeks post-infection (Fig. 2E; median survival time: undefinable); 78042 also showed low virulence with 80% moribundity at 4 weeks, which did not increase further over an additional 3 weeks (Fig. 2F). Despite the poor filamentation of these strains *in vitro*, they were able to cause disease but, as previously described (7), to differing extent.

Based on the behavior of conditionally regulated *NRG1* derivative SC5314 (13), we hypothesized that the *nrg1*∆∆ mutants might increase the virulence of the poorly filamenting strains by promoting filamentation or hypha-associated gene expression. This hypothesis appears to be partially correct. Deletion of *NRG1* in the least virulent 94015 strain did not affect virulence in a statistically significant manner, although there was a trend toward increased virulence (Fig. 2E). We examined the fungal burden of four surviving animals from both groups and found that the 94015 strain had been cleared in three of four animals, while all four of the surviving animals infected with *nrg1*∆∆−94015 had robust kidney fungal burden (Fig. S1A). Consistent with our initial hypothesis, the *nrg1*∆∆−78042 strain had a modest increase in virulence relative to the parental strain (Fig. 2G).

In contrast, deletion of *NRG1* in the two more virulent strains, 57055 and 78048, reduced virulence substantially in both strains (Fig. 2F and H). The *NRG1* mutants of both strains caused reduced fungal burden at day 3, with the difference between the *nrg1*∆∆−78048 and 78048 being statistically significant (Fig. 2B and D). Thus, it is possible that the reduced initial fungal burden of these mutants contributes to their lower virulence relative to the corresponding parental strains. Interestingly, the fungal burden of the kidneys of surviving mice infected with either *nrg1*∆∆−57055 or *nrg1*∆∆−78048 was 10^5^ and 10^6^ CFU/mL, respectively (Fig. S1B and C). The reduced virulence of these two strains, therefore, is not due to clearance of the fungus. This indicates that loss of *NRG1* function does not drive increased virulence in all strains.

### Deletion of *NRG1* in poorly filamenting clinical isolates increases pseudohypha formation *in vitro*

**Fig 3:**
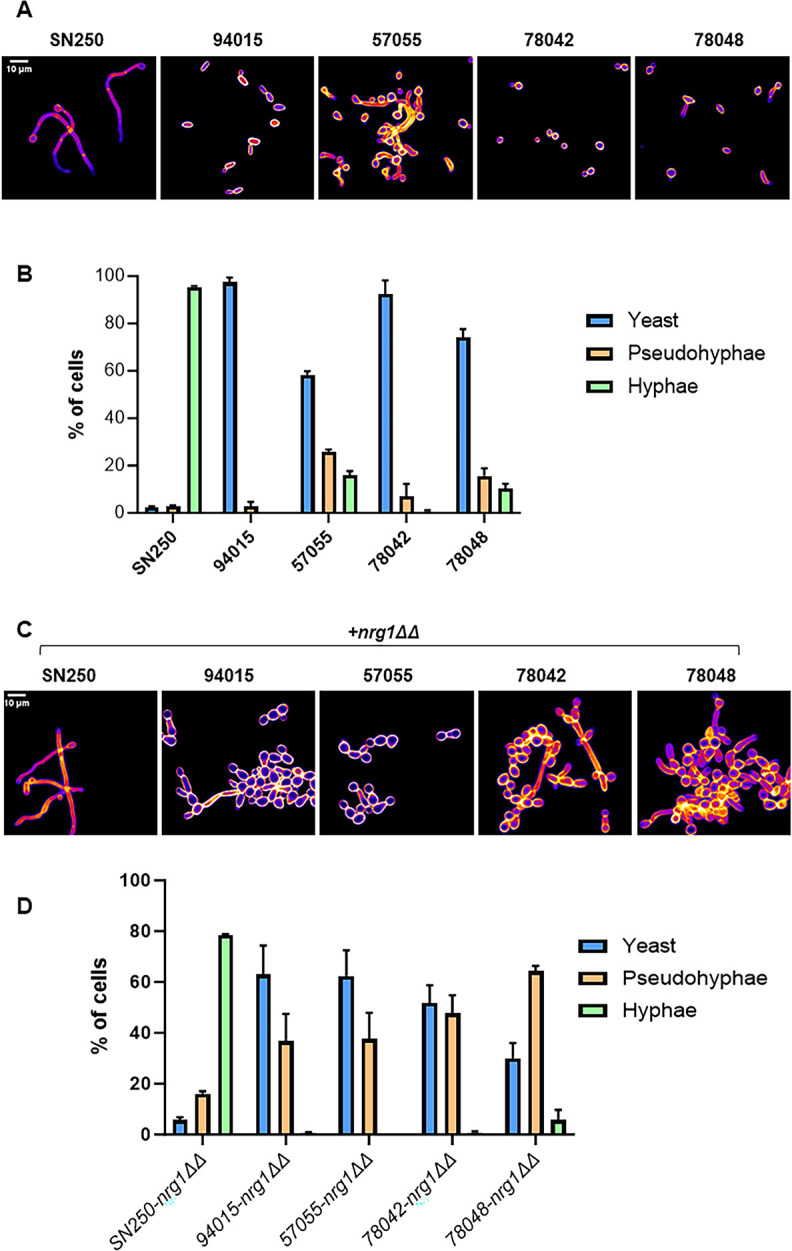
Clinical strains show low rates of filamentation *in vitro*, and deletion of *NRG1* leads to increased pseudohyphae. (**A**) Representative images of indicated strains after incubation in RPMI + 10% bovine calf serum for 4 h at 37°C and calcofluor white staining. (**B**) Quantitation the yeast, pseudohyphal, and hyphal forms in the *nrg1*∆∆ mutants in the indicated strain backgrounds. The bars indicate the mean of three independent experiments with at least 100 cells counted in multiple microscopy fields. Error bars are standard deviations. (**C**) Representative images of *nrg1*∆∆ mutants in the indicated strain background after incubation in RPMI + 10% bovine calf serum for 4 h at 37°C and calcofluor white staining. (**D**) Quantitation the yeast, pseudohyphal, and hyphal forms in the *nrg1*∆∆ mutants in the indicated strain backgrounds. The bars indicate the mean of three independent experiments with at least 100 cells counted in multiple microscopy fields. Error bars are standard deviations.

Hirakawa et al. had found that none of the four clinical isolates formed significant filaments when induced with RPMI tissue culture medium for 6 h at 37°C (4). We tested their ability to filament in RPMI + 10% bovine calf serum (BCS) at 37°C for 4 h to determine if the addition of serum increased their filamentation. For all strains, yeast remained the predominant morphology under these conditions (Fig. 3A and B). All strains formed some filamentous forms with pseudohyphae outnumbering true hyphae and 94015 forming very low numbers of pseudohyphae (Fig. 3A and B). Thus, the addition of serum did not greatly affect the *in vitro* phenotypes and did not provide insights into differences in virulence between the different strains.

Exposure of the *nrg1*∆∆ mutants to filament-inducing conditions led to pseudohypha formation in all strain backgrounds (Fig. 3C and D). The 94015-*nrg1*∆∆ and 57055-*nrg1*∆∆ mutants formed ~2:1 ratio of yeast to pseudohyphae with no hyphae observed. The 78042-*nrg1*∆∆ mutant formed a 1:1 ratio of yeast to pseudohyphae, while the 78048-*nrg1*∆∆ mutant formed predominantly pseudohyphae with a small number of true hyphae observable. The predominance of pseudohyphae in these *nrg1*∆∆ mutants is distinct from the SN250-*nrg1*∆∆ mutant, which forms 80% hyphae (Fig. 3D). Although deletion of the repressor of filamentation *NRG1* in these low filamenting clinical isolates increases filamentation relative to their parental strains under inducing conditions, the strains form pseudohyphae instead of true hyphae, indicating that their inability to form true hyphae is unlikely to be due to a failure to inhibit Nrg1 repression under filament-inducing conditions.

We also examined the filamentation phenotypes of the clinical isolates and their corresponding *NRG1* deletion mutants on solid media (RPMI and RPMI + 10% BCS). The only two parental strains that showed significant filamentation at either 30°C or 37°C were SN250 and 78048 (Fig. S2B and C, respectively). In all strains, the *NRG1* deletion mutants formed highly wrinkled colonies at both 30°C and 37°C in both serum-free and serum-containing media (Fig. S2B). Peripheral invasion was evident at 30°C for all mutants in RPMI (but not RPMI + 10% BCS) and was robust in the SN250 and 78048 derivatives; however, this invasion was lost in all but the SN250- and 78048-*nrg1*∆∆ mutants at 37°C. Thus, during filamentation on solid medium, the colony morphology phenotypes of *nrg1*∆∆ mutants are reasonably consistent across this set of four strain backgrounds.

Taken together, the *in vitro* filamentation phenotypes of both the parental and the *nrg1*∆∆ mutants are fairly consistent across the four clinical isolates. Thus, it is not possible to explain the differences in virulence of either the parental or *nrg1*∆∆ mutants using these phenotypes. It is, however, very clear that, in contrast to *nrg1*∆∆-SN250, the clinical isolate-derived *nrg1*∆∆ mutants form pseudohyphae rather than hyphae under inducing conditions. It appears that Nrg1 suppresses filamentation in these clinical isolates but that the formation of hyphae is dependent upon additional factors that are absent or are not responsive to *in vitro* filamentation cues in the poorly filamenting clinical isolates relative to SN250.

### *In vivo* filamentation phenotypes of the clinical isolates and their *nrg1*∆∆ mutants are strain dependent

**Fig 4:**
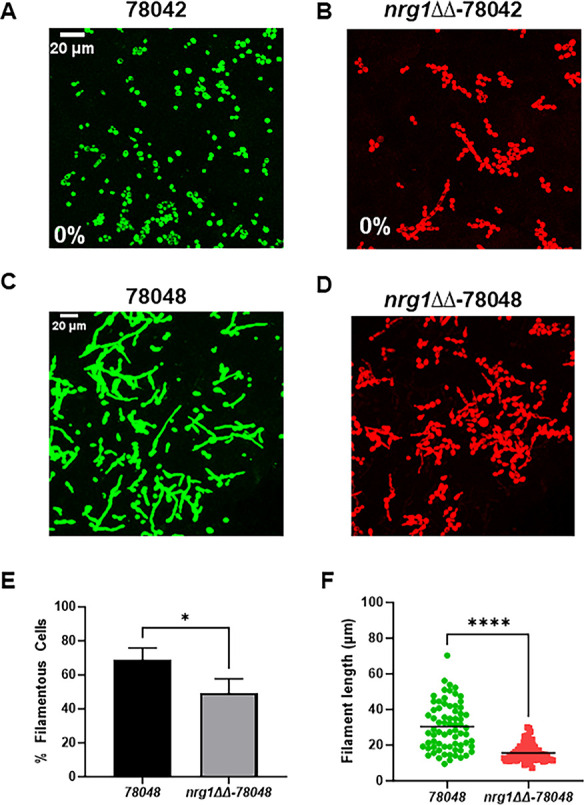
Effect of *NRG1* deletion on *in vivo* filamentation in clinical strains 78042 and 78048. The indicated strains were injected into the subdermal ear tissue of DBA/7 mice and observed 24 h post-infection by confocal microscopy. Representative images for mNEON-78042 (**A**), iRFP-*nrg1*∆∆−78042 (**B**), mNEON-78048 (**C**), and iRFP-*nrg1*∆∆−78048 (**D**) taken 24 h post-infection of ear tissue as described in Materials and Methods. For strain 78042, no filamentous cells were observed as indicated by the 0% in the image. (**E**) Quantitation of % filamentous cells for strain 78048 using the scoring criteria described in Materials and Methods. The bars indicate at least two independent replicates with standard deviation indicated by error bars. *P < 0.05 by Student’s *t*-test. (**F**) Filament length for the 78048 strain dots indicate individual filaments with data pooled from two independent infections. ****P < 0.00001 by Mann-Whitney *U* test.

We next asked if the virulence properties of the clinical isolates correlated with their ability to filament *in vivo*. To do so, we used our *in vivo* imaging model of *C. albicans* filamentation (9). In this model, fluorescently labeled *C. albicans* spp. are directly injected into the subdermal tissue of the mouse ear and quantitatively characterize the morphology of the cells using confocal microscopy at 24 h. In a previous publication, we reported that 94015 failed to undergo filamentation *in vivo* (15), while 50755 formed filaments to an extent similar to SN250 and SC5314 despite having reduced filamentation under a variety of *in vitro* conditions [Fig. 3A and B (9)]. Therefore, poor *in vivo* filamentation of 94015 correlates with its low virulence, and robust *in vivo* filamentation correlates with the ability of 57055 to cause disease. To further test this correlation, we examined the *in vivo* filamentation phenotypes of 78042 and 78048. Consistent with the phenotypes for 50755 and 94015, the low-virulence 78042 strain forms essentially no filaments *in vivo* (Fig. 4A), while the virulent 78048 forms filaments (68%, Fig. 4C) at a rate similar to that previously observed for 50755 and SN250 (15). Thus, the *in vivo* filamentation phenotypes of these strains correlate well with their virulence, while the *in vitro* filamentation phenotypes do not.

We focused our *in vivo* filamentation analysis of the *nrg1*∆∆ mutants on 78042, a poorly filamenting strain *in vivo*, and 78048, a robustly filamenting strain *in vivo*. Despite showing increased virulence relative to its parental strain, the *nrg1*∆∆−78042 strain remained in the yeast morphology *in vivo* with no clearly filamentous forms identified (Fig. 4B). Strikingly, the *nrg1*∆∆−78048 strain showed a statistically significant reduction in the proportion of filaments and a significant shortening of the filament lengths relative to its parental strain (Fig. 4D through F). Although it is not possible to conclusively distinguish hyphae and pseudohyphae with this *in vivo* imaging assay, pseudohyphae have shorter filament lengths. Thus, the *nrg1*∆∆−78048 mutant may be forming more pseudohyphae relative to the parental strain or may be forming shorter hyphae, a feature also correlated with reduced virulence (15). Therefore, the reduced virulence observed for the *nrg1*∆∆−78048 mutant may be due in part to reduced filamentation. On the other hand, the increased virulence of the *nrg1*∆∆−78042 strain cannot be attributed to increased filamentation.

### *In vitro* expression of hypha-associated genes is reduced in poorly filamenting clinical isolates relative to SN250 and increases in their *NRG1* deletion mutants

**Fig 5:**
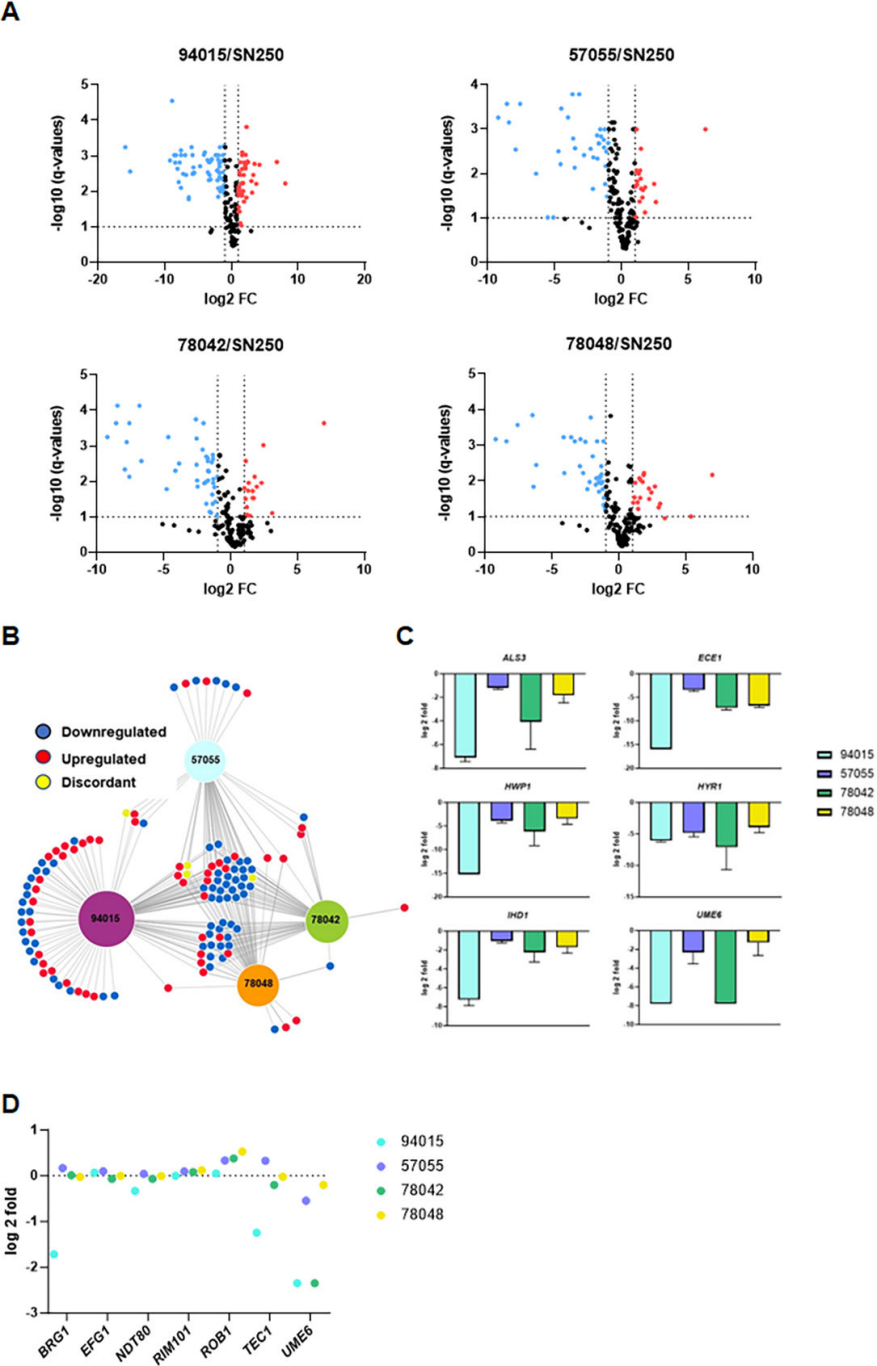
Poor filamenting clinical isolates show reduced expression of hypha-specific genes *in vitro*. (**A**) Volcano plots of gene expression of a set of 185 genes as characterized by NanoString nCounter. The expression was normalized to the strongly filamenting reference strain SN250. The horizontal bar indicates an FDR of 0.1 (Benjamini-Hochberg), and the vertical line indicates log_2_ = 1 of the fold change, which is the cutoff value for the definition of differentially expressed genes. (**B**) Network diagram showing differentially expressed genes that are unique or common between the different clinical strains. The size of the hubs for each clinical isolate is proportional to the number of differentially expressed genes relative to SN250. (**C**) Expression of representative hypha-induced genes for the indicated strains normalized to expression in SN250. Bars indicate the mean fold change of three independent replicates, and error bars indicate standard deviations. (**D**) Log_2_ fold change for the expression of the indicated hypha-associated transcription factors in the four clinical isolates normalized to SN250.

A well-characterized set of hypha-associated genes is induced during filamentation, and some directly contribute to virulence (e.g., *ECE1*). Thus, changes in the expression of hypha-associated genes could contribute to differences in virulence for the clinical isolates and their respective *nrg1*∆∆ mutants. To explore this further, we first characterized the expression profile of the clinical isolates by NanoString under *in vitro* induction and compared the profiles to the strongly filamenting SC5314-derivative SN250. The probe set contains 186 environmentally responsive genes including 57 hypha-associated transcripts (see Table S1 for complete gene list); as this is not a genome-wide set of genes, this limits our conclusions to a focused set of genes related to the function of Nrg1 during filamentation and, thus, not to its other functions. Volcano plots showing the fold change (FC) in expression relative to SN250 for each clinical strain are shown in Fig. 5A; the numbers of differentially expressed genes [DEGs; log_2_ FC ± 1 with a false discovery rate (FDR) of 0.1; Benjamini-Hochberg procedure] for each clinical isolate were 94015 (42 up, 63 down); 57055 (18 up, 35 down); 78042 (17 up, 42 down); and 78048 (20 up, 37 down).

As shown in Fig. 5B, 94015 had the largest set uniquely downregulated genes, which is likely related to its lack of a functional Efg1 allele (4). In contrast, the other three strains had very similar sets of downregulated genes. Twenty-three genes were downregulated in all four strains and included hypha-associated genes; representative examples of these are shown in Fig. 5C. Finally, *YWP1* is expressed during yeast phase growth and is repressed during hyphal growth (16); all clinical strains showed increased expression of *YWP1* relative to SN250 (Table S1). Thus, the expression profile of the poorly filamenting strains is consistent with their *in vitro* filamentation phenotype.

Lack of a functional *EFG1* allele provides a mechanism for the inability of 94015 to undergo filamentation (4). Therefore, one possible mechanism for the reduced filamentation of the other strains is reduced expression of the other transcription factors (TFs) that are required for *in vitro* filamentation relative to SN250. With the exception of 94015, the expression of *BRG1*, *EFG1*, *NDT80*, *RIM101*, *ROB1*, and *TEC1* did not differ significantly from SN250 (Fig. 5D). Efg1 activates the expression of *BRG1* and *TEC1* during filamentation (15), and these TF genes were expressed at much lower levels in 94015. *UME6* is a TF required for the maintenance phase of filamentation as well as a marker of the filament expression program because it is not expressed at appreciable levels until filamentation is initiated (17). *UME6* expression is undetectable in yeast and remains so in 94015 as well as in 78042 during *in vitro* filamentation (Fig. 5D); consistent with this observation, these two strains form almost no hyphae or pseudohyphae. In contrast, *UME6* expression is expressed at levels near that of SN250 in 57055 and 78048, the two strains which form the highest levels of filaments *in vitro*; however, SN250 forms hyphae, while 57055 and 78048 form pseudohyphae. This suggests that *UME6* expression is not sufficient to drive hyphae formation in these strains and that alteration in other aspects of the hyphal program is likely responsible for the lack of hyphae formation in 57055 and 78048.

We also generated NanoString expression profiles for the *nrg1*∆∆ deletion mutants during *in vitro* filament induction (see Fig. S3A through D for volcano plots for each mutant compared to its parental strain with the number of differentially expressed genes shown in each figure; see Table S1 for raw, processed, and FC values along with FDR for each comparison). Although the clinical strain-derived *nrg1*∆∆ mutants do not form true hyphae like the SN250-derived mutants, we expected they might express hypha-associated genes at higher levels. The total numbers of DEGs in the *nrg1*∆∆ deletion mutants of the clinical strains were 49015 (total 96: 56 up, 34 down); 57055 (total 62: 39 up, 23 down); 78042 (total 39: 30 up, 9 down); and 78048 (total 33: 25 up, 8 down).

**Fig 6:**
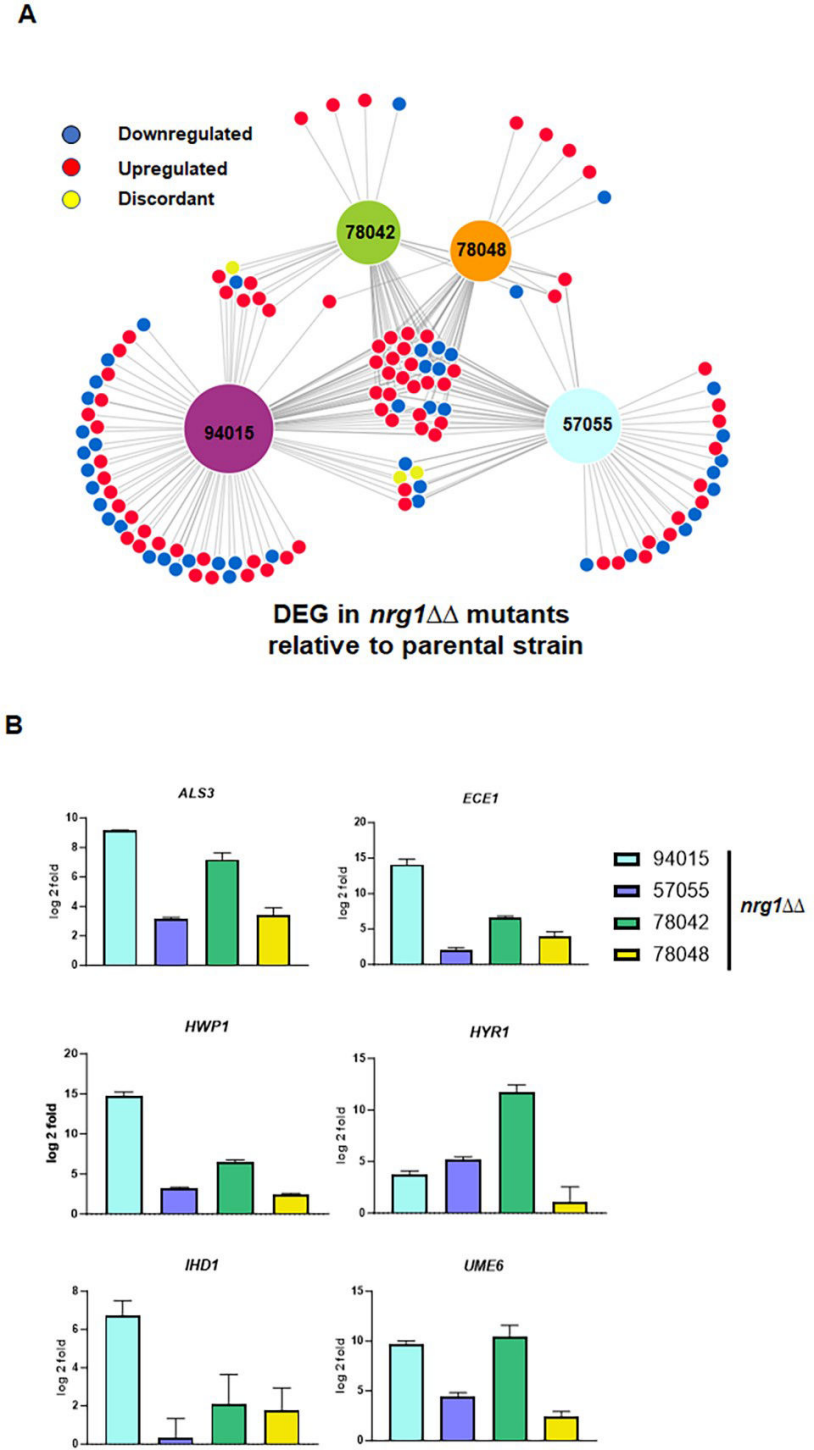
Deletion of *NRG1* increases expression of hypha-associated genes in the clinical isolate backgrounds *in vitro*. (**A**) Network diagram showing differentially expressed genes in the *nrg1*∆∆ mutants relative to the corresponding parental strains. The size of the hubs for each clinical isolate is proportional to the number of differentially expressed genes in the *nrg1*∆∆ relative to its parent. (**B**) Expression of representative hypha-induced genes for the *nrg1*∆∆ mutant normalized to expression in its corresponding parental strain. Bars indicate the mean fold change of three independent replicates, and error bars indicate standard deviations.

Thirty-five genes are differentially expressed in a concordant manner in all four *nrg1*∆∆ mutants (Fig. 6A). This set includes hypha-associated genes that are upregulated in all backgrounds as shown in Fig. 6B. We previously found that deletion of *NRG1* in an *efg1*∆∆ mutant (SN250 background) restored expression of hypha-associated genes *in vivo* and *in vitro* (15). Consistent with the previous observations, hypha-specific gene expression is increased *nrg1*∆∆−94015 (Fig. 6B), even though the strain does not form hyphae *in vitro* (Fig. 3C). The *nrg1*∆∆−94015 strain has the largest set of unique differentially expressed genes among the four strains. The sets of differentially expressed in the *nrg1∆∆*−78042 and *nrg1∆∆*−78048 are almost completely shared with each other and the other two *NRG1* mutants (Fig. 6A). These data are consistent with results from the Mitchell lab, indicating that the effect of transcriptional regulators on gene expression shows both similarities and distinctions across different strain backgrounds (5, 6).

### Strain-dependent differential expression of hypha-associated *in vivo* correlates with the effect of *nrg1*∆∆ mutation on virulence of 78042 and 78048 strains

**Fig 7:**
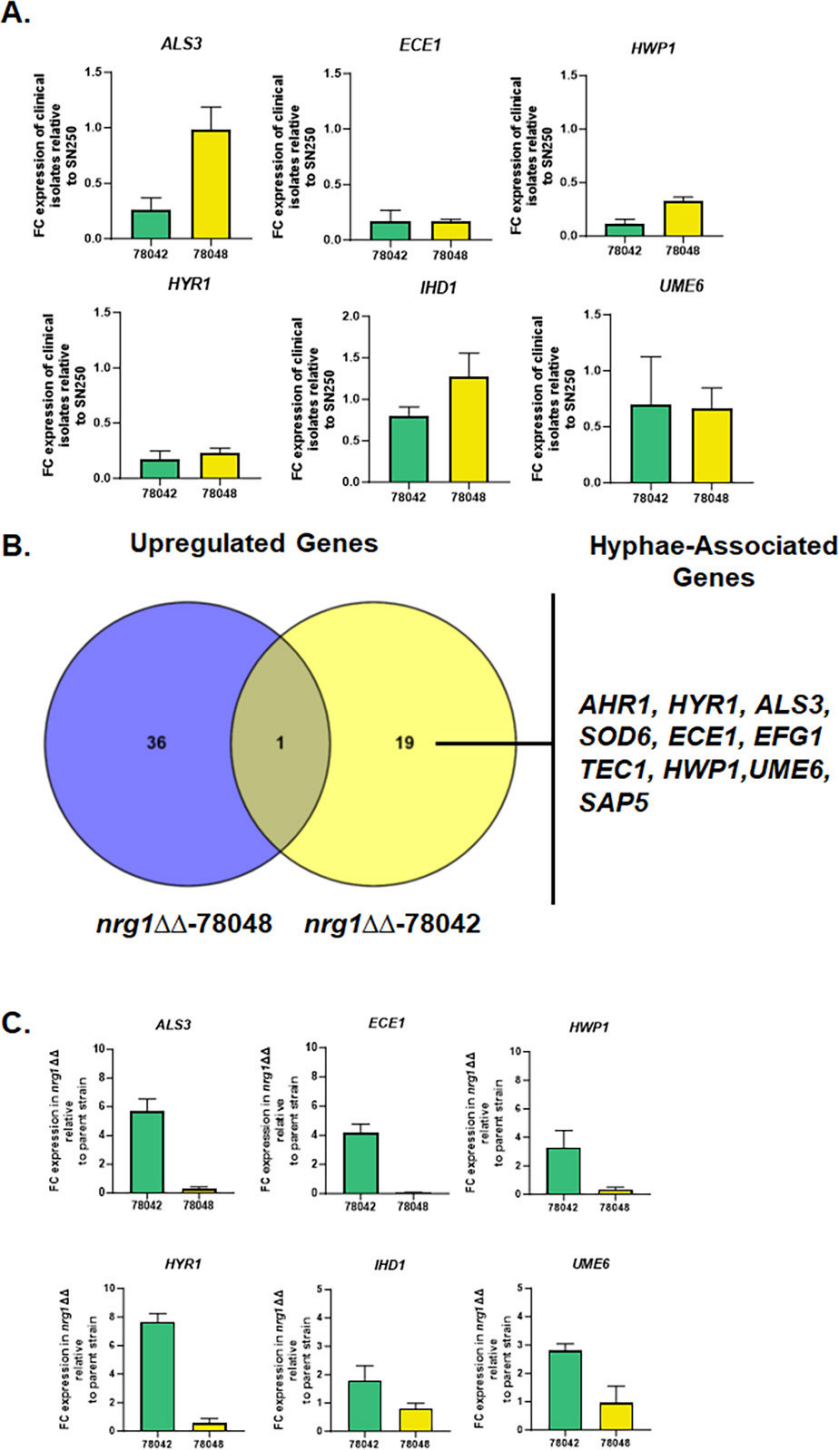
*In vivo* expression profiles of clinical isolates 78042 and 78048 as well as their respective *nrg1*∆∆ mutants 24 h post-infection in an ear model. (**A**) The expression for six hypha-associated genes *in vivo* in 78042 and 78048 compared to the strongly filamentous strain SN250 in the ear model. Bars indicate FC relative to SN250 for three independent replicates. RNA was harvested as described in Materials and Methods 24 h post-infection. *Statistically significant change relative to SN250 (±2-fold change in expression, FDR <0.1). (**B**) Venn diagram of genes upregulated during infection in *nrg1*∆∆−78042 relative to 78042 and *nrg1*∆∆−78048 relative to 78048. See Tables S1 and S2 for FC data and significance data. (**C**) Expression of the indicated hypha-associated genes in *nrg1*∆∆−78042 and *nrg1*∆∆−78048 normalized to expression in the parental strain. Bars indicate FC relative in the *nrg1*∆∆ mutant relative to the parental strains for three independent replicates. RNA was harvested as described in Materials and Methods 24 h post-infection. *Statistically significant change relative to the parental change (±2-fold change in expression, FDR <0.1).

To determine if alterations in *in vivo* gene expression could contribute to, or partially explain, the virulence phenotypes observed with these strains and their *nrg1*∆∆ mutants, we performed NanoString analysis of ear tissue 24 h post-infection with 78042, 78048, and their corresponding *nrg1*∆∆ mutants (see Fig. S3A through D for volcano plots for each mutant compared to its parental strain; see Table S2 for raw, processed, and FC values along with FDR for each comparison). Compared to SN250 (15), the expression of some hypha-associated genes was reduced in 78042 and 78048 *in vivo* (Fig. 7A). Consistent with 78042 forming very few, if any, filaments, the expression of hypha-associated genes was reduced compared to the robustly filamentous 78048. In contrast, 78048 forms the same proportion of filaments as SN250 *in vivo* [Fig. 4 (15)], but the expression of hypha-associated genes is significantly reduced, indicating that a similar level of hyphal morphogenesis does not result in similar levels of hypha-associated gene expression *in vivo*. For example, the expression of candidalysin (*ECE1*), a key virulence-associated toxin (18), is 10-fold lower in 78048 compared to SN250 (Fig. 7A), but the strain has a median survival time and fungal burden similar to those of SN250 [Fig. 2D and H (14)].

Next, we compared the *in vivo* expression profiles of the *nrg1*∆∆ mutants to the 78042 and 78048 parental strains (see Fig. S4A and B for volcano plots and Table S2 for raw and processed data). As shown in the Venn diagram in Fig. 7B, the sets of upregulated genes in the two *nrg1*∆∆ mutants were remarkably different. Surprisingly, only a single gene was upregulated in both *nrg1*∆∆−78042 and *nrg1*∆∆−78048 strains *in vivo*. Furthermore, the set of upregulated genes in the *nrg1*∆∆−78048 strain contained no hypha-associated genes, while multiple key hypha-associated genes were upregulated in the *nrg1*∆∆−78042 strain including the positive transcriptional regulators of hyphae morphogenesis *AHR1*, *EFG1*, *TEC1*, and *UME6* (Fig. 7B; Table S2). The expressions of selected hypha-associated genes in *nrg1*∆∆−78042 and *nrg1*∆∆−78048 are shown in Fig. 7C.

Notably, upregulation of these positive regulators of *in vivo* and *in vitro* morphogenesis does not lead to significant changes in the amount of *in vivo* filamentation observed in the *nrg1*∆∆−78042 mutant relative to its parental strain. Loss of *NRG1* does, however, trigger the increased expression of multiple hypha-associated genes including *ECE1*, *HWP1*, and *HYR1* (Fig. 7C). Consistent with the upregulation of hypha-associated genes in the *nrg1*∆∆−78042 strain, the yeast-associated gene *YWP1* is downregulated eighfold relative to 78042 (Table S2). Consequently, loss of *NRG1* in 78042 de-represses the expression of hypha-associated genes but does not lead to a corresponding increase in filamentation. The expression data further suggest that the increase in virulence observed for the *nrg1*∆∆−78042 strain relative to 78042 may be due to increased expression of hypha/virulence-associated genes such as *ECE1* or *HWP1*.

In contrast, deletion of *NRG1* in 78048 does not increase the expression of hypha-associated genes and, in fact, leads to the reduced expression of two canonical hypha-associated genes, *ECE1* and *ALS3* (Fig. 7C). Again, this observation fits with the reduced filamentation of the *nrg1*∆∆−78048 strain *in vivo*. *ECE1* expression is reduced 6-fold in 78048 compared to SN250 and is further reduced by 16-fold in the *nrg1*∆∆−78048 mutant. Thus, it seems possible that the dramatically reduced expression of *ECE1* in the *nrg1*∆∆−78048 mutant may also contribute to its reduced virulence (19), although reduced filamentation or reduced expression of other genes could also play a role.

## DISCUSSION

Using a set of clinical isolates with relatively poor *in vitro* filamentation, we explored the relationship between *in vitro* and *in vivo* filamentation and virulence. From this analysis, we can draw three conclusions. First, *in vitro* filamentation phenotypes using standard conditions, including host-like conditions, correlate with neither infectivity nor virulence in the four isolates that we examined. This supports the conclusion of a previous study using historical virulence data (4, 7). Second, the two isolates (94015 and 78042) that do not filament *in vivo* are less virulent than the two strains (57055 and 78048) that filament *in vivo*. These findings suggest that at least some of the discordance between *in vitro* filamentation phenotypes and *in vivo* virulence is due to the fact that the infection environment drives filamentation in some strains that do not undergo filamentation well *in vitro*. Third, *C. albicans* strains that are unable to filament *in vitro* or *in vivo* (94015 and 78042) are still able to establish infection in the kidney, the major target organ of this model. This is consistent with previous observations that yeast-locked mutants of SC5314 also establish kidney infection (13).

At the outset of the project, we somewhat naively hypothesized that deletion of *NRG1* would increase the filamentation of these poorly filamenting strains and potentially increase their virulence as well. *In vitro*, deletion of *NRG1* leads to a consistent phenotype in which the strains show increased pseudohypha formation and increased expression of hypha-associated genes. Although there are some strain-specific distinctions in the DEG sets, these are generally minor with the exception of *nrg1*∆∆−94015, which lacks Efg1. Thus, at a broad level, the *in vitro* function of Nrg1 is consistent across multiple clinical strains.

There are, however, phenotypic differences between *nrg1*∆∆-mutants in the clinical isolates and the SN250-derived mutant. First, as previously described by many others (10, 11), deletion of *NRG1* in SC5314-derivatives leads to constitutive pseudohypha formation in the absence of hypha-inducing stimuli, whereas the yeast morphology is predominant in *nrg1∆∆* mutants generated in the four poorly filamenting strains. However, this phenotype is one of degree rather than a simple binary state because all four poorly filamenting do form pseudohyphae in non-inducing conditions. Thus, additional factors must be present in SN250 that mediates the strong constitutive pseudohyphal phenotype; the poor filamenting strains apparently lack these factors. Second, upon *in vitro* induction, the *nrg1*∆∆-SN250 forms predominately hyphae, while the clinical isolate-derived *nrg1*∆∆ mutants generate a mixture of yeast and pseudohyphae with almost no true hyphae observed. This further suggests that the mechanisms which make SC5314 and its derivatives robustly filamentous and that are lacking in the clinical isolates operate downstream of the relief of Nrg1 repression step.

It is unclear what genes or pathways drive the robust filamentation of SC5314, although we have recently found that a gain-of-function SNP present in the transcription factor Rob1 contributes to the strong filamentation of SC5314; we have not identified this SNP in any other sequenced strain (14); deletion of *NRG1* in a SC5314-derived mutant with only the more prevalent *ROB1* allele did not block hyphae formation (Wakade and Krysan, unpublished), indicating that this cannot explain the differential effect of *NRG1* deletion between these strains and SC5314. On the other hand, it is still possible that strain-specific alterations in the function of positive regulators of filamentation may be present in these strains. However, an examination of the mutations in 78042 and 78048 (relative to SC5314) did not reveal any obvious alterations in key hypha-related regulators (4). Thus, additional studies will be required to identify the hypha-associated pathways that are lacking in these strains. The increasing availability of diverse *C. albicans* clinical isolates should facilitate that search.

Whereas the *in vitro* phenotypes and expression profiles of the four clinical isolate-derived *nrg1*∆∆ mutants are fairly consistent, they show phenotypic heterogeneity *in vivo*. We initially hypothesized that deletion of *NRG1* would increase virulence in general. However, this hypothesis was confirmed conclusively for only one of the four strains. Strikingly, the increased virulence of the *nrg1*∆∆−78042 strain was not due to increased filamentation but can be explained, at least in part, by the increased expression of hypha-associated genes such as *ECE1* in cells that remained in yeast morphology *in vivo*. Contrary to our hypothesis, deletion of *NRG1* led to reduced virulence in the two most virulent of our four strains (57055 and 78048). This difference was not due to a failure to establish initial infection or due to clearance of the mutants from the kidneys.

In the case of the *nrg1*∆∆−78048 mutant, the reduced virulence is likely to be due to multiple factors including altered extent of filamentation, filament length, and non-filamentation-associated factors. Most notable, however, is the reduced expression of the toxin *ECE1* in the *nrg1*∆∆−78048 mutant relative to the parental strain. *In vitro* and *in vivo*, deletion of *NRG1* increases the expression of *ECE1* in a multiple strain background. *In vitro*, the *nrg1*∆∆−78048 mutant has increased expression of *ECE1*, but *in vivo*, it is reduced by 16-fold. These results indicate that, under some conditions and in some strains, the loss of the repressor Nrg1 leads to decreased expression of some of its canonical targets. These seemingly paradoxical findings are consistent with a similar strain-dependent effect of the *NRG1* deletion reported by the Mitchell lab and highlight the complexity and plasticity of transcriptional networks across different *C. albicans* strains (6).

Finally, 78048 is an α/α strain and could, in principle, switch *in vivo* from white to opaque, leading to either differences in regulation of filamentation or non-filamentation-based virulence traits. Our NanoString probe set contains *WH11*, a gene expressed in white cells; the *in vivo* expression of *WH11* is increased threefold (FDR 0.005, Table S2) in the *nrg1*∆∆−78048 relative to the 78048 parental strain, suggesting that it is remaining in the white phase *in vivo*. It is, of course, important to keep in mind that Nrg1 affects the expression of non-filament-associated genes, and it is possible, and indeed likely, that such affects may also contribute to differences in virulence.

Our characterization of the *in vivo* filamentation and virulence phenotypes of the four parental strains also provides some explanations for the discordance between *in vitro* filamentation and virulence. Specifically, the ability of some strains to undergo filamentation *in vivo* but not *in vitro* is likely to explain some of the phenotypic contradictions. Since some strains that cannot undergo filamentation *in vivo* are less virulent than those that do, we propose that filamentation remains a general virulence trait for *C. albicans*. However, our data also suggest that alteration in the expression of hypha/virulence-associated genes in the absence of strong changes in filamentation is also likely to contribute. It is important to consider that even the strain lacking a functional allele of the master virulence and filamentation regulator Efg1 caused disease in these models and, since it was isolated from the blood of a patient, in humans to some extent. As such, we cannot forget the role of the host and the reasons or risk factors that predisposed them to invasive candidiasis.

One of the questions that motivated this set of experiments was “is deletion of the filament repressor *NRG1* sufficient to increase filamentation in clinical isolates with low rates of filamentation?” Our results clearly show that, for some strains, this is not sufficient and that additional factors are required for robust filamentation such as is observed for the reference strain SC5314. *C. albicans* is a commensal of the gastrointestinal (GI) tract in humans, and strains with reduced filamentation are more successful colonizers of the GI tract (20). Thus, one advantage of low-filamenting *C. albicans* strains is increased fitness as a colonizer of the GI tract. In principle, a strain could become a more successful colonizer by maintaining Nrg1 repression or limiting its de-repression. If this were the primary mechanism for the reduced filamentation of some of the strains we studied, then deletion of *NRG1* would restore filamentation. Indeed, this was observed by Lemberg et al. in the case of oral isolate 101, indicating that this successful colonizer may have reduced Nrg1 de-repression (21). Our data, however, indicate that strains with reduced filamentation and virulence also arise through variation in the activation of filamentation pathways downstream of Nrg1. *C. albicans* appears to employ a variety of genetic strategies as it adapts to niches in the human host.

## MATERIALS AND METHODS

### Strains, cultivation conditions, and media

The *C. albicans* clinical isolate strains (94015, 57055, 78042, and 78048) used in this study were obtained from the Soll laboratory (7). Standard recipes were used to prepare yeast peptone dextrose (YPD) (22), and all *C. albicans* strains were precultured overnight in YPD medium at 30°C. RPMI medium was purchased and supplemented with bovine serum (10%, vol/vol). For *in vitro* hyphal induction in liquid media, *C. albicans* strains were incubated overnight at 30°C in YPD media, harvested, and diluted into RPMI + 10% serum at a 1:50 ratio and incubated at 37°C for 4 h (9). For plate-based filamentation assays, the precultured strains were diluted to 0.1 Optical Density (OD_600_) in water and spotted (5 µL) on YPD or RPMI + 10% BCS agar plates. The plates were dried, incubated at either 30°C or 37°C for 3 days, and photographed. Images are representative of two independent replicates.

### Strain construction

*C. albicans* transformation was performed using the standard lithium acetate transformation method (23). The homozygous mutant strains of *C. albicans* were constructed using the transient CRISPR/Cas9 method (23). Oligonucleotides and plasmids used to generate the mutant strains in this study are listed in Table S3. The *NAT1-Clox* system (24) was used to generate the *NRG1* KO strains. Briefly, both copies of the *NRG1* were deleted by amplifying *NAT1-Clox* cassette from *NAT1-Clox* plasmid with primer pairs *NRG1.P1* and *NRG1.P2* and by using sgRNA targeting both alleles of the *NRG1* gene. The resultant transformants were selected on YPD containing 2.5-mM methionine and 2.5-mM cysteine, along with 200-µg/mL nourseothricin (Werner Bioagents, Jena, Germany). The correct transformants were confirmed by standard PCR methods with primer pairs *NRG1.P5* and *NRG1.P6*. Oligonucleotide sequences are provided in Table S3.

Fluorescently labeled strains were generated by using p*ENO1-NEON-NAT1* and p*ENO1-iRFP-NAT1* plasmids as previously described (9), and the resultant transformants were selected on YPD containing 200-µg/mL nourseothricin (Werner Bioagents). The reference strain was tagged with green fluorescent protein (NEON), whereas the mutant strains were tagged with iRFP.

### *In vitro* characterization of *C. albicans* morphology

Induced cells were fixed with 1% (vol/vol) formaldehyde. Fixed cells were then imaged using the Echo Rebel upright microscope with a ×60 objective. The assays were conducted in triplicate on different days to confirm reproducibility.

### Mouse model of disseminated candidiasis

Five- to six-week-old female CD1 mice (Envigo) were inoculated with 1 × 10^6^ CFU (200 µL) of reference or mutant strain by lateral tail-vein injection. A group of five mice was used to assess the kidney fungal burden. Mice were monitored daily and were sacrificed 3 days after infection as per the Institutional Animal Care and Use Committee (IACUC) guidelines to determine the kidney fungal burden. Two kidneys were homogenized together in 1-mL sterile phosphate-buffered saline (PBS); 10 µL of 10-fold serial dilutions of kidney extracts was spotted on to the YPD plates; and fungal burden was determined with unpaired *t*-test.

To determine the virulence of the reference or mutant strains, 10 female CD1 mice per group were inoculated with the 1 × 10^6^ CFU (200 µL) by lateral tail-vein injection. Mice were monitored daily. When a mouse exhibited any signs of extreme morbidity such as hunched back, head tilt or tremors, the mouse was euthanized as per the IACUC guidelines. Survival curves were plotted on a Kaplan-Meier curve, and log-rank (Mantel-Cox) test was used to determine the statistical difference of the curves (GraphPad Prism).

### *In vitro* and *in vivo* RNA extraction

RNA extractions for the *in vitro* and *in vivo* samples were carried our as described previously (15). Briefly, for *in vitro* RNA extractions, three independent samples were grown in YPD at 30°C, washed twice in the PBS, diluted at 1:50 ratios in the RPMI + 10% serum, and incubated at 37°C for 4 h. Cells were collected, centrifuged for 2 min at 11,000 rpm at room temperature (RT) and RNA was extracted as per the manufacturer protocol (MasterPure Yeast RNA Purification Kit, Cat. No. MPY03199). Extraction of RNA from mouse ear (two to three biological replicates) was carried out as described previously (15). Briefly, 24 h post-infection, the mouse was euthanized following the protocol approved by the University of Iowa IACUC. The *C. albicans* injected mouse ear was excised and placed in an ice-cold RNAlater solution. Subsequently, the ear was transferred to a mortar, flash-frozen using liquid nitrogen, and ground to a fine powder. The powder was collected into a 5-mL centrifuge tube, and 1 mL of ice-cold Trizol was added. The samples were placed on a rocker at RT for 15 min and then centrifuged at 10,000 rpm for 10 min at 4°C. The cleared Trizol was then collected without dislodging the pellet into a 1.5-mL Eppendorf tube, and 200 µL of RNase free chloroform was added. The tubes were shaken vigorously for 10–15 s and kept at RT for 5 min. Furthermore, the samples were centrifuged at 12,000 rpm for 15 min at 4°C. The cleared aqueous layer was then collected to a new 1.5-mL RNAase-free Eppendorf tube, and RNA was further extracted using the Qiagen RNeasy kit protocol.

### NanoString analysis of gene expression *in vitro* and *in vivo*

NanoString analysis was carried out as described previously (15). Briefly, in total, 80 ng of *in vitro* or 400 ng of *in vivo* RNA was added to a NanoString codeset mix and incubated at 65° C for 18 h. After hybridization reaction, samples were processed using an nCounter prep station and were scanned on an nCounter digital analyzer. nCounter .RCC files for each sample were imported into nSolver software to determine the quality control metrics. Using the negative control probes, we defined the background values and used these as a background threshold, and this value was subtracted from the raw counts. The resulting background subtracted total raw RNA counts were first normalized against the highest total counts from the biological triplicates (two to three for all experiments) and then to the highest total counts for the samples. The statistical significance of changes in gene expression was determined using the Benjamini-Hochberg method at an FDR of 0.1. The raw, normalized, and analyzed expression data are provided in Tables S1 and S2. The data sets were deposited at the NCI GEO repository under accession number GSE253732.

### *In vivo* imaging and filamentation scoring

The inoculation and imaging of mice ear were carried out as described previously (9, 15, 25). Acquired multiple Z stacks (minimum 15) were used to score the yeast vs filamentous ratio. The cells were considered as a “yeast” if the cells were round and/or budded. Furthermore, yeast cells were required not to project through multiple Z stacks. The cells were considered as “filamentous” if the cells contain intact mother and filamentous which was at least twice the length of the mother body. A minimum of 100 cells from multiple fields were scored. Paired Student’s *t*-test with Welch’s correction (*P* > 0.05) was used to define the statistical significance, which was carried out using GraphPad Prism software. The filament lengths of the *in vivo* samples were measured as described previously (15, 23). Briefly, a Z stack image of the reference or mutant strain was opened in an ImageJ software, and the distance between the mother neck to the tip of the filament was measured. At least 50 cells per strain from multiple fields were measured. Statistical significance was determined by Mann-Whitney *U* test (*P* > 0.05).

## SUPPLEMENTAL MATERIAL

Fig. S1 msphere.00785-23-s0001.tif Kidney fungal burden.

Fig. S2 msphere.00785-23-s0002.tif Solid agar plate filamentation.

Fig. S3 msphere.00785-23-s0003.tif Volcano plots for *in vivo* expression data.

Fig. S4 msphere.00785-23-s0004.tif Volcano plots for *nrg1* mutants *in vivo*.

Legends msphere.00785-23-s0005.docx Captions for supplemental figures and tables.

Table S1 msphere.00785-23-s0006.xlsx *In vitro* expression data.

Table S2 msphere.00785-23-s0007.xlsx *In vivo* expression data.

Table S3 msphere.00785-23-s0008.xlsx Oligonucleotides.

ASM does not own the copyrights to Supplemental Material that may be linked to, or accessed through, an article. The authors have granted ASM a non-exclusive, world-wide license to publish the Supplemental Material files. Please contact the corresponding author directly for reuse.
