# Supplementary figures and images for: The role of the C. albicans transcriptional repressor NRG1 during filamentation and disseminated candidiasis is strain dependent
Source: mSphere. 2024 Feb 20;9(3):e00785-23. doi: 10.1128/msphere.00785-23 (PMC10964420; doi:10.1128/msphere.00785-23)

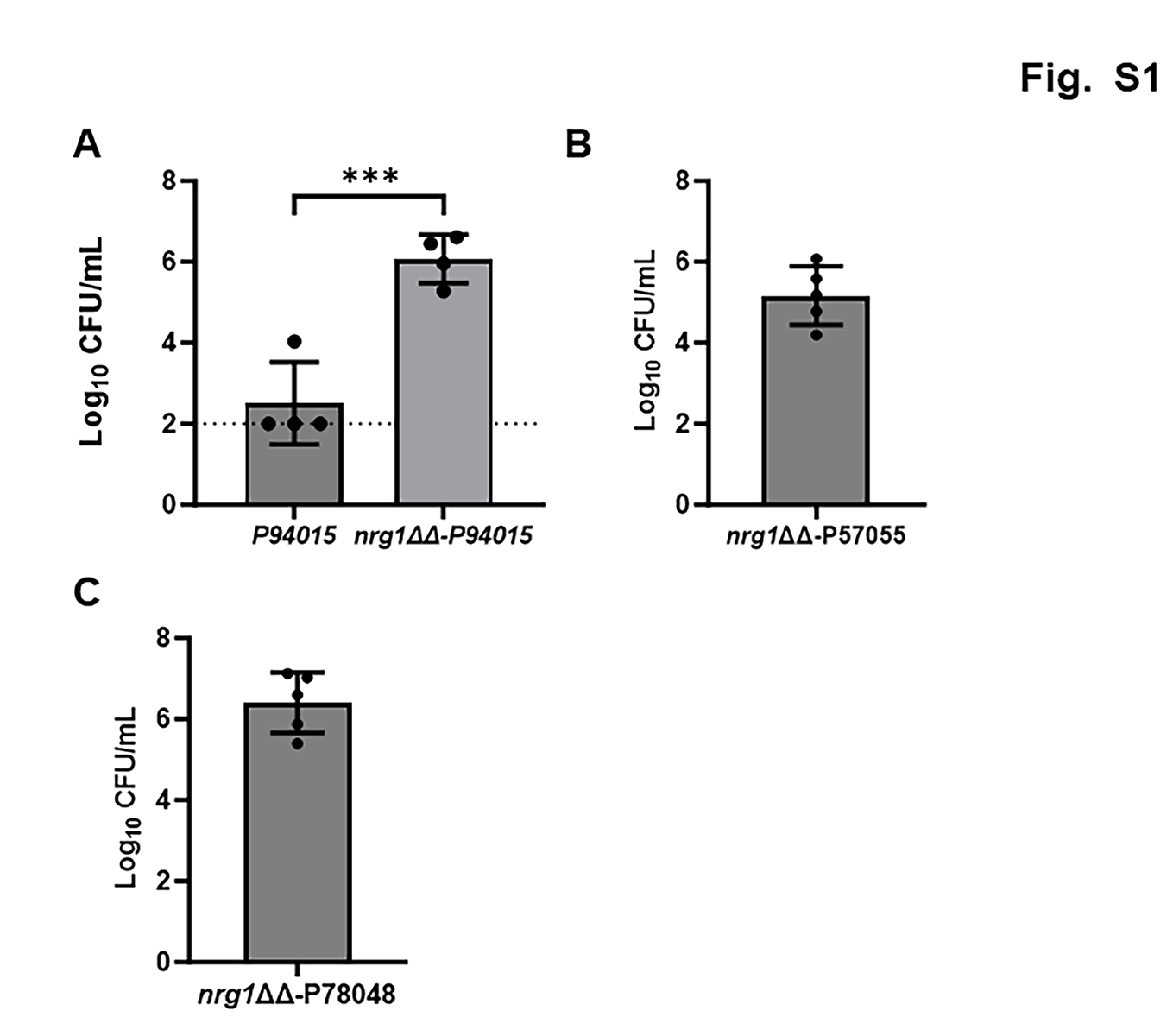

Supplement: Fig. S1 — Kidney fungal burden. [file msphere.00785-23-s0001.tif]

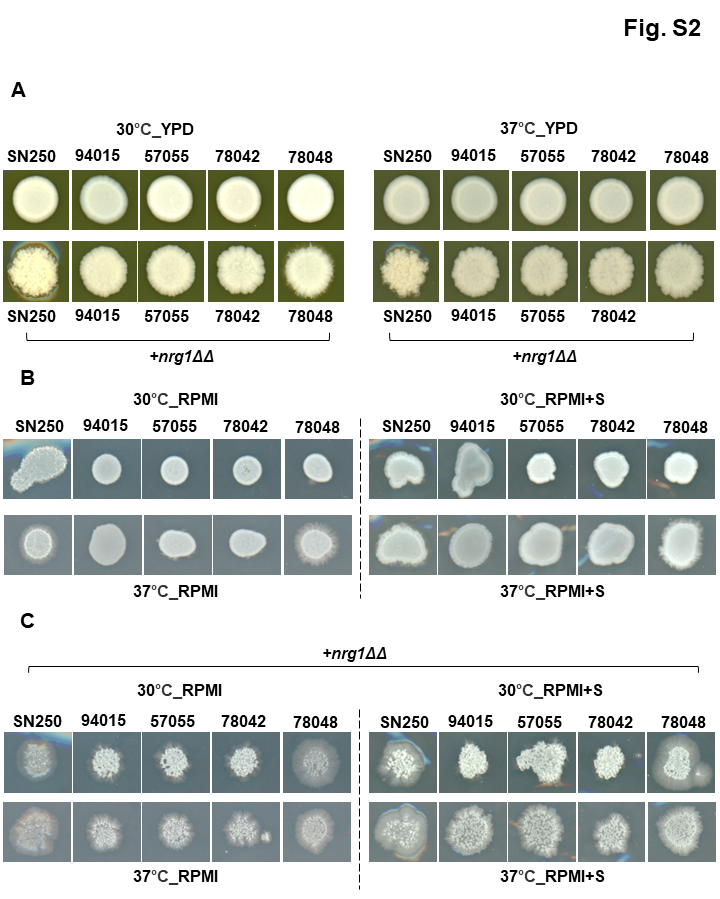

Supplement: Fig. S2 — Solid agar plate filamentation. [file msphere.00785-23-s0002.tif]

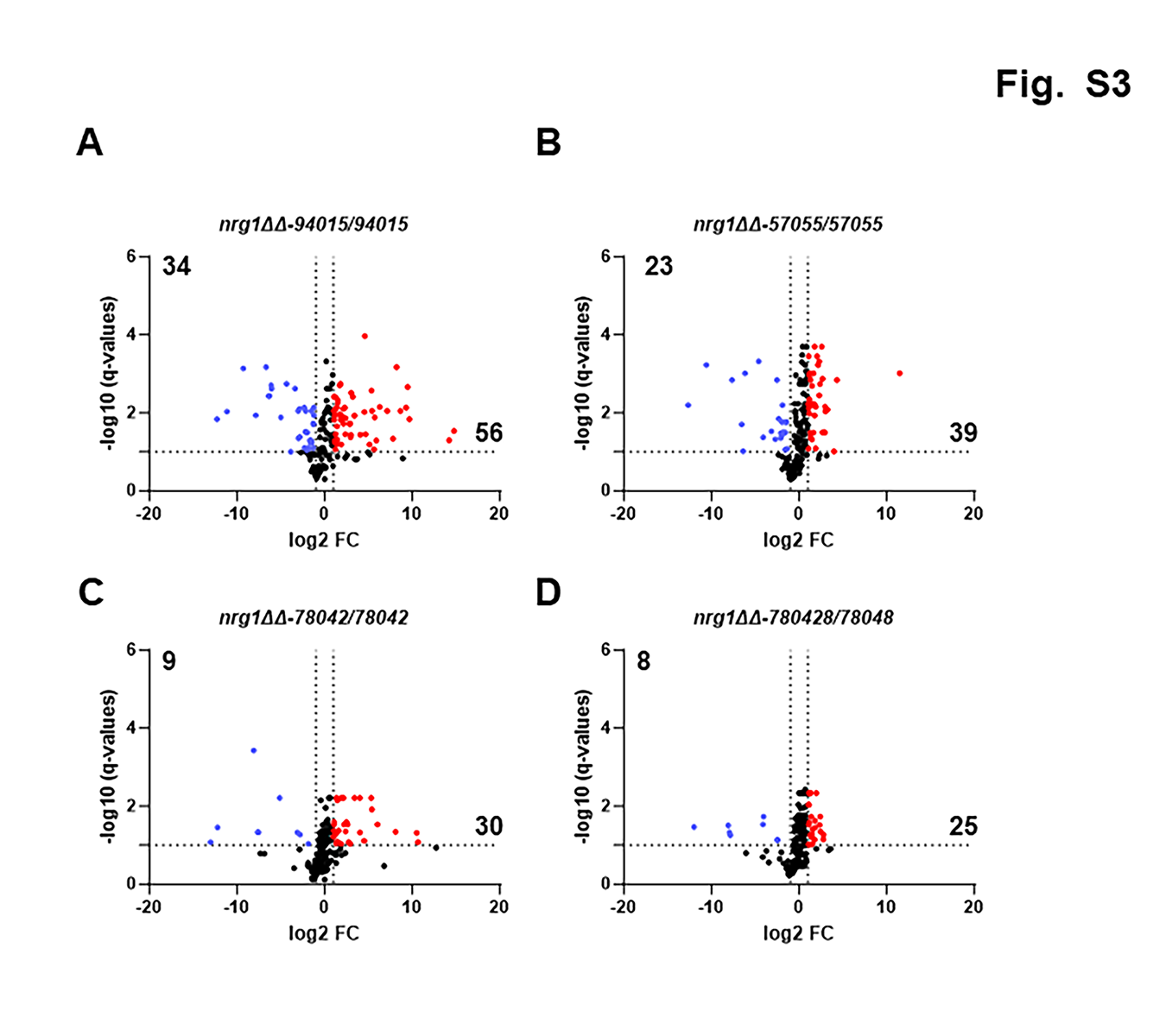

Supplement: Fig. S3 — Volcano plots for in vivo expression data. [file msphere.00785-23-s0003.tif]

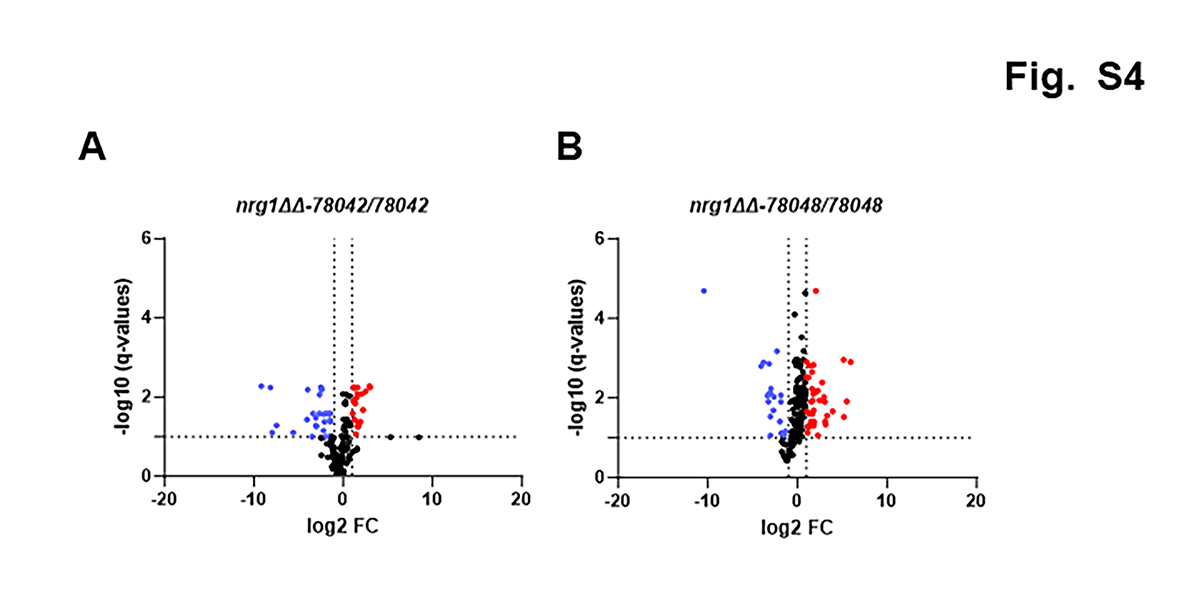

Supplement: Fig. S4 — Volcano plots for nrg1 mutants in vivo. [file msphere.00785-23-s0004.tif]
