# Supplementary material for: The role of the C. albicans transcriptional repressor NRG1 during filamentation and disseminated candidiasis is strain dependent
Source: mSphere. 2024 Feb 20;9(3):e00785-23. doi: 10.1128/msphere.00785-23 (PMC10964420; doi:10.1128/msphere.00785-23)
Supplement: Legends — Captions for supplemental figures and tables. [file msphere.00785-23-s0005.docx]

**Supplementary Materials**

**Fig. S1. Kidney fungal burden for surviving animals in virulence experiments.** Kidney fungal burden from animals surviving at the end of virulence assays (**Fig. 2**). **A**. 94015. **B**. *nrg1*∆∆-57055. **C**. *nrg1*∆∆-78048. Bars indicate mean of log_10_ fungal burden (CFU/mL) with individual mice shown as points and error bars indicating standard deviation. Differences between groups were analyzed by Student’s t test (NS: p> 0.05 and *** p< 0.0001).

**Fig. S2. Filamentation phenotypes of clinical isolates and their corresponding *nrg1*∆∆ mutants on agar plates.** The indicated clinical isolates and corresponding *nrg1*∆∆ mutants were plated on YPD (**A**), RPMI (**B**) and RPMI+10% bovine calf serum (**C**) and incubated at either 30^o^C or 37^o^C for 3 days and photographed. The experiments were performed in biological duplicate and phenotypes were similar in both experiments.

**Fig. S3. Volcano plots comparing the in vitro expression profiles for clinical isolates to their corresponding *nrg1*∆∆ mutants**. Volcano plots of gene expression of a set of 185 genes as characterized by Nanostring nCounter. The expression was normalized to the strongly filamenting reference strain SN250. The horizontal bar indicates FDR 0.1 (Benjamini-Hochberg) and the vertical line indicates log_2_ = 1 of the fold change which are the cutoff values for the definition of differentially expressed genes. **A.** 94015; **B.** 57055. **C.** 78042. **D.** 78084.

**Fig. S4.** **Volcano plots comparing the in vivo expression profiles for clinical isolates 78042 and 78048 to their corresponding *nrg1*∆∆ mutants.** These expression profile data were obtained from strains infected into the ear as described for in vivo imaging. Volcano plots of gene expression of a set of 185 genes as characterized by Nanostring nCounter. The expression was normalized to the strongly filamenting reference strain SN250. The horizontal bar indicates FDR 0.1 (Benjamini-Hochberg) and the vertical line indicates log_2_ = 1 of the fold change which are the cutoff values for the definition of differentially expressed genes. **A.** 78042. **B.** 78084.

**Table S1. Nanostring expression data for in vitro experiments**. RNA was harvested from the indicated strains 4hr after induction with RPMI+10%BCS. Raw mRNA counts, normalized counts, average counts, fold-change from reference strain, Student t test, and Benjamini-Hochberg adjusted FDR are provided for each gene and condition (two to three biological replicates). Differentially expressed genes were defined as ±2-fold-change in expression with FDR<0.1. Fold-change values shown in red are significantly downregulated and those shown in green are significantly upregulated. Summaries of all differentially expressed genes are also provided.

**Table S2. Nanostring expression data for in vivo experiments**. RNA was isolated from mouse ear tissue 24hr post-infection. Raw mRNA counts, normalized counts, average counts, fold-change from reference strain, Student t test, and Benjamini-Hochberg adjusted FDR are provided for each gene and condition (two to three biological replicates). Differentially expressed genes were defined as ±2-fold change in expression with FDR<0.1. Fold-change values shown in red are significantly downregulated and those shown in green are significantly upregulated. Summaries of all differentially expressed genes are also provided.

**Table S3. Oligonucleotides used for strain construction and analysis.**
